# Recent US Gastric Cancer Prevention Recommendations: Advances, Unanswered Questions, and the Need for a Unified Approach

**DOI:** 10.1053/j.gastro.2025.04.012

**Published:** 2025-04-30

**Authors:** DUCO T. MÜLDER, JAMES F. O’MAHONY, MONIKA LASZKOWSKA, JENNIFER YEH, CHIN HUR, IRIS LANSDORP-VOGELAAR, THE CISNET-GASTRIC GROUP

**Affiliations:** Department of Public Health, Erasmus Medical Center, Rotterdam, the Netherlands; Department of Public Health, Erasmus Medical Center Rotterdam, the Netherlands*, and* School of Economics, University College Dublin, Dublin, Ireland; Gastroenterology, Hepatology, and Nutrition Service, Department of Subspecialty Medicine, Memorial Sloan Kettering Cancer, Center, New York, New York; Department of Pediatrics, Harvard Medical School, Boston Children’s Hospital, Boston, Massachusetts; Division of General Medicine, Department of Medicine, Columbia University Irving Medical Center, New York, New York; Department of Public Health, Erasmus Medical Center, Rotterdam, the Netherlands

## Abstract

In the past year, two major American gastroenterology societies have introduced pivotal updates to their recommendations for gastric cancer (GC) prevention.^1–3^ The American College of Gastroenterology (ACG) released new guidelines on *Helicobacter pylori* management, recommending screen-and-treat to individuals at high-risk for GC,^1^ as well as guidelines on the management of gastric precursor lesions.^2^ Moreover, the American Gastroenterological Association (AGA) issued a clinical practice update (CPU) focusing on endoscopic screening and surveillance of high-risk groups for GC.^3^ These updates represent important advances in the rapidly evolving landscape of GC prevention. However, key differences in focus — between *H pylori* screen-and-treat vs endoscopic screening — may leave health care providers uncertain which to prioritize and which populations to consider for these interventions. In this commentary, we aim to provide a perspective on GC prevention integrating these recommendations, highlighting the unanswered questions they raise, and proposing new research avenues.

## Updated GC Prevention Recommendations

Although population-level interventions were previously deemed unnecessary in low GC incidence regions, persistent disparities, the preventable nature of GC, and the success of screening in high-risk regions have sparked interest in GC prevention, even in low-risk settings such as the United States. Recent ACG and AGA updates mark significant steps forward in GC prevention, focusing on different aspects of risk reduction (Table 1). One aspect of the ACG guidelines focuses on primary prevention of GC through eradication of *H pylori*.^1^ Although focused on treatment regimens for *H pylori*, the recommended indications for test-and-treat carry important implications for GC prevention. Unlike previous more restrictive guidelines,^4^ the ACG now recommends *H pylori* test-and-treat for asymptomatic individuals from high-risk demographic groups. Although a quantitative risk threshold is not provided, specified high-risk groups include immigrants from high-risk countries and non-White racial and ethnic groups, in addition to people with premalignant lesions, a family history, or hereditary syndromes. The AGA CPU focuses on similar high-risk groups, but centers on secondary prevention of GC through endoscopic screening.^3^ The update suggests starting screening at the age of 45 years to coincide with colorectal cancer (CRC) screening, though optimal starting and stopping ages remain uncertain. Endoscopic surveillance is recommended every 3 years, depending on the findings at index endoscopy and other risk factors. While acknowledging *H pylori* eradication, it suggests that this approach should be an adjunct to endoscopic screening. Finally, the ACG guidelines on management of premalignant conditions suggest against general endoscopic screening and state that recommendations on screening in high-risk groups cannot be made, focusing instead on effective opportunistic diagnosis and surveillance.

## Current Evidence for GC Prevention

The updated recommendations come at a time when the evidence for both primary and secondary prevention strategies is largely derived from high-risk regions. The ACG’s recommendations reflect systematic reviews of trials, primarily conducted in East-Asia, demonstrating the effectiveness of *H pylori* eradication in reducing GC incidence.^5^ The population benefits of *H pylori* eradication are also supported by observational evidence from large-scale eradication programs in Taiwan.^6^ Although Western regions lack the same wealth of data generated in Asia, observational registry studies in the United States also demonstrate that *H pylori* eradication reduces GC burden.^5,7^ Furthermore, modeling studies show that *H pylori* screen-and-treat may be cost-effective in Western countries, despite lower incidence.^8^ Despite concerns about potential increases in antibiotic resistance, this growing evidence base has supported the adoption of *H pylori* screen-and-treat recommendations in Western guidelines, as reflected in the ACG recommendations and recent European Union policy recommendations.^1,9^

Similar to the evidence for *H pylori* screen-and-treat, endoscopic screening is supported by data from Asia, with established screening programs in Japan and South Korea,^10^ and large-scale trials conducted in China.^11^ In the United States, evidence demonstrating population-level benefits of endoscopic screening remains limited and the balance between harms and benefits is unclear. As such, AGA CPU guidelines cautiously advise that endoscopic screening should be considered for selected high-risk groups rather than endorsing it for widespread implementation. ACG guidelines recommend against endoscopic screening in the general population and state they are unable to make recommendations for high-risk groups due to insufficient direct evidence from US populations.

Although the evidence base is still evolving, there is an urgent need for effective GC prevention policies in the United States. Rising incidence rates in certain demographic groups underscore the persistent racial and socioeconomic disparities in the GC burden.^12^ Moreover, it is concerning that GC ranks last (19^th^/19^th^) in terms of the National Cancer Institute funding-to-lethality ratio,^13^ particularly given that most cases are attributable to a treatable risk factor and largely avoidable.

## Unanswered Critical Questions

The limited availability of robust evidence has prompted caution with respect to recommendations, leaving several critical questions unanswered. Should prevention efforts focus on endoscopic screening, *H pylori* screen-and-treat strategies, or a combination? What is the optimal age to screen? What is the optimal interval for surveillance? How should high-risk groups be defined, and at what points do the benefits outweigh the harms of the interventions? What are the broader implications of these strategies for health care? Addressing these knowledge gaps is essential for the implementation of effective prevention.

### The Focus of GC Prevention: H pylori Screen-and-Treat and/or Endoscopy?

It is crucial to recognize that *H pylori* screen-and-treat strategies and endoscopic screening are interdependent. Similar to human papilloma-virus vaccination and cervical cancer screening,^14^ a high uptake of *H pylori* screen-and-treat interventions may change the balance between harms and benefits of endoscopy by reducing the likelihood of detecting lesions. Despite this interdependence, the existing guidelines have focused on addressing primary and secondary prevention as siloed approaches, rather than comprehensively considering the harms and benefits of integrated strategies. The ACG guidelines do not advise endoscopic screening in GC prevention in the general population and do not provide conclusive guidance on screening high-risk groups. The AGA practice update frames *H pylori* test-and-treat “as an adjunct to endoscopic screening” but the exact recommended relationship between these modalities remains unclear. While the term “adjunct” might imply that eradication is intended for patients with detection of *H pylori* during endoscopy, the guidelines also mention eradication at younger ages and familial eradication, hinting at a broader role. Overall, these guidelines do not provide clarity on how *H pylori* screen-and-treat strategies and endoscopic screening and surveillance strategies should be integrated, leaving providers uncertain about the optimal approach to reduce GC risk and mortality for their high-risk individuals.

Age may play a critical role in defining the optimal strategy. Because the greatest benefit of *H pylori* eradication is achieved before the development of gastric precursor lesions, screen-and-treat may be most effective in younger populations.^15^ For older cohorts in which precursor lesions may already be prevalent and potentially irreversible, the impact of eradication could be limited. In these cohorts, endoscopic screening may provide an opportunity for early detection and intervention in individuals who missed *H pylori* eradication efforts earlier in life.

Endoscopy in high-risk populations could also be considered as an adjunct to *H pylori* eradication rather than the reverse. For individuals with a confirmed *H pylori* infection at older ages, an endoscopic evaluation could identify the presence of premalignancies. This dual approach, currently absent from existing recommendations, may ensure a targeted intervention for those at greatest risk. Moreover, race, ethnicity, and immigrant status as GC risk factors are likely due to association with higher *H pylori* prevalence, thus more widespread testing could reduce the reliance on racial/ethnic factors as proxies for disease risk. Given the ethical challenges of screening according to race and ethnicity may pose,^16^ finding more precise metrics for disease risk, as stated in the AGA CPU, is warranted.^3^

In Europe, societies for endoscopy, pathology, and the study of *Helicobacter* and gut microbiota have collaborated to develop a combined guideline aimed at providing a unified approach for GC prevention.^17^ In contrast, the difference in focus between the recent updates of the American recommendations emphasizes certain outstanding questions and uncertainties about the relative roles of *H pylori* eradication and endoscopic screening in GC prevention. Whether one strategy should take precedence or if they should be implemented in tandem may depend on multiple factors, such as the individual disease risk, health care resource availability, and patient preferences. Further studies on the comparative effectiveness of *H pylori* screen-and-treat vs endoscopy screening, and how this may differ by age, are needed to integrate these strategies.

### What Constitutes High Risk?

Both recommendations highlight similar characteristics to select high-risk populations for screening (Table 1). However, neither set of recommendations clarify at what specific risk threshold these benefits become greater than the harms. This is critical, given the substantial variation in risk levels within the defined high-risk groups. For example, Korean American individuals (with an incidence of 49.0 per 100,000) and non-Hispanic Black individuals (11.2 per 100,000) both fall into the high-risk category but have a nearly five-fold risk-difference.^18^ Additionally, development of risk calculators incorporating factors beyond race could help identify more specific groups at even higher risk. While our commentary focuses on US-based guidelines, a previous review of endoscopy guidelines also demonstrated scope for global harmonization on which populations merit index endoscopic screening.^19^

Differences in GC risk among potential screening populations have major implications for the balance between benefits and harms. The AGA CPU makes an elegant comparison between GC and CRC incidence to suggest that GC risk in some demographic groups may be comparable to CRC, for which screening is widely accepted.^3^ While this comparison is informative, a detailed quantification of the benefits (such as the prevented GC mortality) and harms (such as the number of required tests) for populations with varying risk levels would be valuable. Such studies could clarify the point at which the benefits of screening outweigh the harms.

In addition to identifying characteristics that define high-risk populations for screening, the ACG clinical guideline on premalignant lesions and the AGA CPU also outline precursor lesions that warrant surveillance. These are based on the extent and histologic subtype, as also specified in previous practice updates.^20^ Just as defining a risk threshold is important for screening initiation, further guidance is needed on the exact risk-stratification algorithm and interval for surveillance. Cohort studies tracking the yield of endoscopic surveillance of patients with premalignancies, along with modeling studies, could facilitate a favorable balance between harms and benefits.

### Impact on Health Care Beyond GC

Mass eradication of *H pylori* and endoscopic screening have far-reaching consequences for health and health care beyond GC. One major concern with *H pylori* eradication is the potential effect of antibiotic use on antibiotic resistance. Although the ACG guidelines outline strategies for managing antibiotic resistance through adjustments in treatment and susceptibility testing, the broader effects of increased antibiotic use in screen-and-treat interventions on resistance remain unclear. Increased use may raise resistance rates, especially for ACG-recommended antibiotics such as rifabutin, which appears on the WHO antibiotic watchlist for high resistance risk.^21^ Given that antibiotic resistance can influence treatment success not only for *H pylori*, but also for other bacterial infections, the consequences for health care could be substantial. However, Taiwanese data show negligible long-term resistance increases after mass eradication.^6^ Further investigation of the interplay between *H pylori* screen-and-treat and antibiotic resistance is therefore needed. Such studies should include longitudinal surveillance to track resistance rates in populations implementing screen-and-treat programs, and infectious disease modeling to predict the broader impact of antibiotic use on resistance patterns. Another concern is the potential link between *H pylori* eradication and esophageal adenocarcinoma. However, a large-scale Nordic cohort-study found no elevated risk, although ongoing monitoring may still be warranted.^22^ Despite these concerns, eradication may yield benefits beyond GC, such as reducing the burden of peptic ulcer disease and dyspepsia. These potential positive effects should also be factored into recommendations.

The implementation of endoscopic screening has significant implications for health care capacity. Most notably, it requires a substantial increase in the number of endoscopies demanded. This highlights the need for training endoscopists, particularly given the varying quality of upper-endoscopies and relatively high miss rate for GC during endoscopy (10%).^23^ Additionally, the need for biopsies during endoscopy adds to pathologists’ workload and costs. Endoscopic screening may also result in an initial rise in cancer incidence by detecting prevalent cancers, as observed following the introduction of Korea’s national screening program.^24^ Health care facilities must be prepared for these additional patients, making resource planning essential for effective implementation.

### Decision Analysis Modeling for Unanswered Questions

Despite the urgency for effective GC prevention policy, clinical studies in the United States are unlikely to provide all necessary data due to challenges regarding required sample sizes and follow-up durations. Decision modeling can play a critical role in integrating evidence to answer important research questions. A recent expert working group report from the International Agency for Research on Cancer highlighted the role of such models in GC prevention.^25^ However, because of limited availability of data on the unobservable development of precancerous lesions, models inherently carry uncertainty. Such uncertainty could partially be addressed by comparative modelling, such as through initiatives like the Cancer Intervention and Surveillance Modeling Network (CISNET).

## Conclusions

The ACG guidelines on *H pylori* and management of gastric premalignant lesions, and the AGA CPU on GC screening and surveillance are bold steps towards action. However, further integration of endoscopy and *H pylori* screen-and-treat recommendations is needed to maximize population benefits and guide public health policy in the United States. The current evidence supporting *H pylori* screen-and-treat interventions appears more abundant than that for endoscopic screening. Therefore, we suggest its use in carefully selected populations at substantially higher risk, particularly at younger ages, as a strong starting point. However, criteria regarding which population subgroups should be prioritized must be further clarified. Additionally, concerns regarding antibiotic resistance must further be investigated, especially if screen-and-treat interventions are expanded to broader high-risk sub-populations, such as all non-Hispanic Black and Asian individuals. Although the benefits of *H pylori* test-and-treat have been consistently demonstrated, the evidence regarding the balance of risks and benefits for endoscopic screening remains less established, particularly in low-risk regions such as the United States. Given these limitations, a cautious approach remains appropriate. Ultimately, clinicians should engage in shared decision-making with patients to ensure personalized care.

Optimal GC prevention policy requires continuous monitoring of current interventions and global collaboration, particularly between scientific societies of regions with comparable risk profiles. Screening initiatives therefore benefit from structured organization and implementation through pilot studies, enabling systematic monitoring of harms and benefits. A pragmatic approach for such studies may be to leverage existing CRC screening infrastructure. Data from these studies, combined with our knowledge on the natural history of GC and data from screening programs in Asia, can help to optimize these interventions. In the current absence of clinical trial studies in the United States, comparative decision modeling studies may further aid in unraveling these vital questions.

## Figures and Tables

**Table 1.** Overview of the Recently Updated Recommendations on GC Prevention

	ACG clinical guidelines		AGA clinical practice update on screening and surveillance in individuals at increased risk for GC in the United States^3^	
	Management of *H pylori*^1^	Management of premalignant conditions^2^		
Screening modality	*H pylori* screen-and-treat	Endoscopic screening	Endoscopic screening	Nonserologic *H pylori* screening as an adjunct to endoscopic screening
Target population for screening^a^	Immigrants from high GC risk countries Certain non-White racial/ethnic groups Current or history of precursors (eg, atrophic gastritis, intestinal metaplasia, dysplasia) History of GC or GC resection Individuals with gastric adenomas or hyperplastic polyps Family history of GC in a first-degree relative Individuals with hereditary syndromes Individuals with autoimmune gastritis	NA	Early-generation immigrants from moderate-high risk countries Non-White racial and ethnic groups Family history of GC in a first-degree relative History of *H pylori* infection and at least one other risk factor (smoking, high-salt diet, living in persistent poverty) Individuals with hereditary syndromes	Individuals at increased risk (see endoscopic screening) Adult household members of individuals who test positive for *H pylori*
Recommended screening age	Not reported	NA	Optimal start age unknown but suggested at 45 years for synergistic effects with colorectal screening; stop age based on comorbidity status	Optimal age not established; 20–40 years suggested based on Taipei Global Consensus
Surveillance	NA	Endoscopic surveillance at 3-year intervals in high-risk groups with GIM plus high-risk histology (Incomplete, corpus-extension), family history, foreign-born, or high-risk race/ethnicity	Suggested endoscopic surveillance every 3 years depending on the extent and histology of detected precursor lesions and other risk factors	NA

ACG, American College of Gastroenterology; AGA, American Gastroenterological Association; GC, gastric cancer; GIM, gastric intestinal metaplasia; NA, not applicable.

a The ACG guidelines considered other indications for *H pylori* testing in patients with symptoms and/or disease. As we focus on screening asymptomatic populations, these were excluded here.
